# Exploration for the real causative agents of licorice-induced pseudoaldosteronism

**DOI:** 10.1007/s11418-021-01484-3

**Published:** 2021-01-22

**Authors:** Toshiaki Makino

**Affiliations:** Department of Pharmacognosy, Graduate School of Pharmaceutical Sciences, Nagoya City University, Nagoya, Japan

**Keywords:** Glycyrrhiza, Licorice, Glycyrrhizin, Adverse effects, Pseudoaldosteronism, 18*β*-glycyrrhetyl-3-*O*-sulfate

## Abstract

I investigated the causative agents of licorice-induced pseudoaldosteronism, which is a frequent side effect of Japanese traditional Kampo medicines. Glycyrrhizin (GL), the main ingredient of licorice, is absorbed after being metabolized to glycyrrhetinic acid (GA) by intestinal bacteria, and then metabolized in liver to 3-monoglucuronyl-glycyrrhetinic acid (3MGA). In normal condition, 3MGA is excreted into bile via a multidrug resistance-related protein (Mrp) 2, therefore, 3MGA does not appear in blood circulation. However, under the dysfunction of Mrp2, 3MGA appears in the blood circulation and is excreted into the urine by not glomerular filtration but tubular secretion via organic anion transporter (OAT) 1 and 3. At this time, 3MGA inhibits type 2 11*β*-hydroxysteroid dehydrogenase (11*β*HSD2) in tubular cells to cause pseudoaldosteronism. Since GA is not the substrates of these transporters, GA cannot inhibit 11*β*HSD2 in tubular cells. Therefore, it was considered that 3MGA was the causative agents of licorice-induced pseudoaldosteronism. After that, I isolated and identified three other GL metabolites, 22*α*-hydroxy-18*β*-glycyrrhetyl-3-*O*-sulfate-30-glucuronide (**1**), 22*α*-hydroxy-18*β*-glycyrrhetyl-3-*O*-sulfate (**2**), and 18*β*-glycyrrhetyl-3-*O*-sulfate (**3**) from the urine of Mrp2-deficient rats orally treated with GA, and found that their blood and urinary concentrations were much higher than 3MGA and that their pharmacokinetic behaviors were similar to 3MGA. 3MGA was not detected in the blood of patients with pseudoaldosteronism who developed rhabdomyolysis due to licorice, and compound **3** was detected at a high concentration. In addition, a multicenter retrospective study was conducted using the serum and urine of 97 patients who took Kampo medicines containing licorice. Of a total of 97 patients, 67 detected GA in the serum (median 122 nM, 5 nM–1.8 µM) and 68 detected compound **3** (median 239 nM, 2 nM–4.2 µM), and there were no cases of detection of GL, 3MGA, compounds **1**, and **2**. High blood concentrations of compound **3** were associated with low plasma renin activity, plasma aldosterone levels, and serum potassium levels. It is highly probable that compound **3** is the true causative agent of pseudoaldosteronism.

## Introduction

Licorice-induced pseudoaldosteronism is a common adverse effect of Kampo medicine. Licorice is registered in United States Pharmacopoeia 43th Edition as the dried roots, rhizomes, and stolons of *Glycyrrhiza glabra* L. or *Glycyrrhiza uralensis* Fish. ex DC. [1], and it is registered as the name of Glycyrrhiza in Japanese Pharmacopoeia Seventeenth Edition (JP XVII) [2]. Licorice is actually utilized in more than 70% of the Kampo formulas approved by the Japanese Medicinal Regulatory Agency, the Ministry of Health Labour and Welfare of Japan [3]. Licorice is used not only as the component of Kampo medicines but also as a natural sweetener for foods and confectionery. In Europe, licorice has been used as a food ingredient for a long time, and it has been reported that ingestion of licorice often causes hypertension and edema. In 1968, this symptom was named as licorice-induced pseudoaldosteronism and came to be recognized as a disease, not just a side effect [4]. Symptoms include hypertension and edema, as well as hypokalemia and hypernatremia with increased potassium secretion in the renal tubules, metabolic alkalosis, hyporeninemia, and myalgia and numbness due to myopathy. Since this condition is sometimes life-threatening [5], its early detection is critical to prevent disease aggravation. Although the frequency with which pseudoaldosteronism caused by Kampo medicines arises depends to some extent on the dosage and duration of licorice treatment [6], its onset exhibits large individual differences and it is generally unpredictable.

**Fig. 1:**
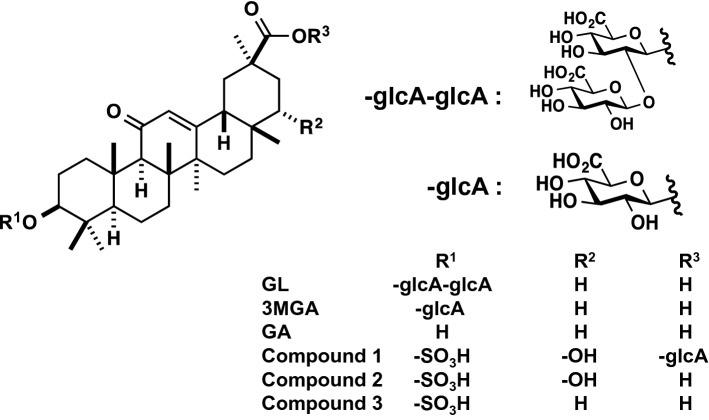
Chemical structures of glycyrrhizin (GL) and its metabolites. GA, 18*β*-glycyrrhetinic acid; 3MGA, 3-monoglucuronyl-glycyrrhetinic acid; compound **1**, 22α-hydroxy-18*β*-glycyrrhetyl-3-*O*-sulfate-30-glucuronide (**1**); compound **2**, 22*α*-hydroxy-18*β*-glycyrrhetyl-3-*O*-sulfate (**2**); compound **3**, 18*β*-glycyrrhetyl-3-*O*-sulfate (**3**)

Licorice contains glycyrrhizin (GL) as the main ingredient (Fig. 1). GL is used not only as an oral preparation for allergy but also as an injection to improve liver function. GL is a glycoside having a structure in which two molecules of glucuronic acid are bound to the hydroxyl group at the 3-position for one molecule of glycyrrhetinic acid (GA), which is the aglycone part. When licorice is orally administered, GL is hard to pass though gastrointestinal epithelium due to the hydrophilicity of sugars, and GA is absorbed as after the sugar part of GL hydrolyzed by the intestinal bacteria inhabiting the large intestine [7]. Therefore, it is considered that the main body of pharmacological activity of licorice is considered to be GA (Fig. 1).

It had long been known that licorice had a corticosteroid-like action, and it was once thought that GL and GA caused pseudoaldosteronism by binding to mineralocorticoid receptors. However, their affinities for the receptor are significantly lower than that of the original substrate, aldosterone, and it was denied that they act by directly binding to the receptor at the actual blood concentration of GL and GA. Cortisol, an adrenocortical hormone, has the same affinity for mineralocorticoid receptors as aldosterone. However, cortisol is decomposed into cortisone by type 2 11*β*-hydroxysteroid dehydrogenase (11*β*HSD2) in the cytoplasm of renal tubule cells, and the receptor is not activated by low affinity of cortisone [8]. GA and GL contained in licorice have the effect of inhibiting 11*β*HSD2, and the inhibitory activity of GA is about 200 times higher than that of GL [9]. In addition, since GL does not appear in the blood when licorice is ingested, it had been considered that GA was the causative agent of the onset of licorice-induced pseudoaldosteronism.

However, the mechanism mentioned above cannot explain the individual differences in the onset of pseudoaldosteronism. In 1995, Kato et al. compared the plasma of patients who had used licorice-containing Kampo medicines or GL preparations between a group who developed pseudoaldosteronism and those who did not, and found 3-monoglucuronyl-glycyrrhetinic acid (3MGA, Fig. 1) in the plasma of the patients who developed pseudoaldosteronism [10]. From this result, it was speculated that the individual constitution of the appearance of 3MGA as a metabolite in blood might be associated with the onset of pseudoaldosteronism.

### Mechanism of 3MGA-induced pseudoaldosteronism

A liver fibrosis rat model was made by using a choline-deficient diet and an examination was made on the pharmacokinetics of GA and 3MGA [11]. In the liver fibrosis model rats, higher plasma 3MGA concentrations were observed than those in normal rats, but there was no difference in the plasma concentration of GA between these groups. In the liver fibrosis model group, the urinary 3MGA excretion was also higher than that in the normal group, but the urinary GA excretion was below the detection limit in both groups. The expression level of multidrug resistance protein 2 (Mrp2), which is a transporter that excretes glucuronic acid conjugates into bile, was lower in liver fibrosis group than that in normal group [11].

Next, I studied the relationship between 3MGA and the onset of pseudoaldosteronism [12]. The half maximal inhibitory concentration (IC_50_) values of 3MGA and GA on 11*β*HSD2 in rat renal microsomes were 0.46 µΜ and 0.13 µΜ, respectively. In rat serum, both 3MGA and GA were present as the binding-form to albumin at a rate of 99.9% or more, so that they are difficult to be excreted by glomerular filtration. Indeed, when normal SD rats orally treated with GA, there are no detection of GA in their urine. When GA was orally administered to Mrp2-deficient Eisai hyperbilirubinuria rats (EHBRs), 3MGA was found in urine, but GA not, suggesting that 3MGA could be excreted by tubular secretion, but that GA not. Since each compound is an anionic compound, I chose organic anion transporter (OAT) 1 and OAT3 that are expressing at the basolateral membrane of renal tubular epithelial cells. Significantly higher amount of 3MGA was imported into HEK293 cells that was temporally expressing OAT1 or OAT3 compared with mock cells, but GA not. It is revealed that not GA but 3MGA is the substrate of OAT1 and OAT3 [12].

From the above results, the following hypotheses can be made regarding the pharmacokinetics after oral administration of GL and the onset of pseudoaldosteronism [13]. When GL is orally administered, GL is hydrolyzed to GA by intestinal bacteria, and then absorbed into blood circulation. GA is not excreted into urine because it cannot pass through glomerular basement membrane by its highly binding to albumin and is not secreted through renal tubules. GA in blood circulation transfers into liver, metabolized to 3MGA by the glucuronate-conjugation, and excreted into bile via Mrp2. In intestine, 3MGA is hydrolyzed to GA again by intestinal bacteria, and is partially absorbed again from the intestine into blood circulation to exhibit enterohepatic circulation, and the unabsorbed portion of 3MGA is excreted in feces. Therefore, if the function of Mrp2 in liver is normal, 3MGA is not present in blood circulation. When bile excretion of 3MGA is suppressed due to Mrp2-dysfunction, 3MGA is transferred into blood circulation. Since 3MGA is also existed in blood circulation with the binding-form to serum albumin, 3MGA is not excreted into urine by glomerular filtration. However, it can be transported from blood circulation into tubular cells via OAT1 and 3, and excreted into urine by tubular secretion. Since 11*β*HSD2 is expressed in tubular cells, it is speculated that not GA but 3MGA can inhibit 11*β*HSD2 to develop pseudoaldosteronism [13].

### Finding other metabolites of GL than 3MGA

Based on the above hypothesis, I considered that the onset of pseudoaldosteronism could be prevented at an early stage by detecting 3MGA in blood or urine after taking licorice. Since licorice is used not only ethical Kampo prescription but over-the-counter drugs, I considered that the detecting kit for 3MGA in blood or urine should be used in drug stores. Therefore, I developed a monoclonal antibody against 3MGA (anti-3MGA-mAb) that can be used for enzyme-linked immunosorbent assay (ELISA) [14]. When the specificity of this antibody to 3MGA was calibrated as 100%, the cross-reactivities to GA and GL were 1.04% and 0.22%, respectively. Using anti-3MGA-mAb, I developed ELISA system to measure 3MGA, a good calibration curve could be created when 3MGA was dissolved in normal rat plasma and urine, and the results of the spike and recovery test were also good.

Next, the 3MGA concentrations in serum and urine samples of Mrp2-deficient EHBRs that orally administered with GA were measured by both LC–MS/MS and ELISA, respectively. However, the observed values of 3MGA concentrations by ELISA were 40–100-fold higher than those measured by using LC–MS/MS, although the profiles were similar to one another, suggesting that the unknown metabolites that can be cross-reacted with anti-3MGA-mAb were existed in serum and urine samples of Mrp2-deficient EHBRs treated with GA.

EHBRs were administered GA via drinking water for 3 months, and their urine was collected and pooled. Then, I conducted eastern blot analysis using anti-3MGA-mAb for the detection of 3MGA and other compounds which have related structure in urine sample. Urine or 3MGA standard solution was spotted onto a TLC plate, developed using the solvent, transferred onto a polyethersulfone (PES) membrane, and fixed. The membrane was treated with anti-3MGA-mAb, enzyme-labeled secondary antibody, and its substrate 4-chloro-1-naphthol. The positive staining spots were appeared other than 3MGA, which position was more hydrophilic than 3MGA.

**Fig. 2:**
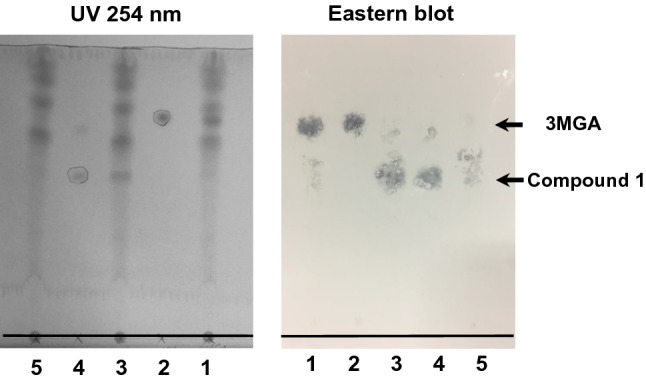
Eastern blot analysis of urine collected from EHBRs orally treated with GA using anti-3MGA mAb [14]. On the photographs, base lines were added. On TLC plate, the following samples were spotted at the base line; lane 1, double spot of 6 μl of urine collected from EHBRs which were orally administered with GA as drinking water (1 mg/ml) and 2 μl of 3MGA (1 mM); lane 2, 2 μl of 3MGA (1 mM); lane 3, double spot of 6 μl of urine collected from EHBRs and 2 μl of compound **1** (1 mM); lane 4, 2 μl of compound **1** (1 mM); lane 5, 6 μl of urine collected from EHBRs. Then, the spots were spreaded out using H_2_O/BuOH/AcOH (2:7:3), and the top line of the solvent was penciled. Photograph of TLC plate detected by UV absorption at 254 nm (left) was taken. On the lanes 2 and 4 used as positive control, the spots appeared were marked by pencil. The spots on the plate were transferred onto PES membrane, fixed, blocked, and strained by anti-3MGA MAb. Photograph of the membrane was taken (right). *Rf* values of 3MGA and compound **1** were 0.76 and 0.50 respectively

From pooled urine collected from EHBRs orally treated with GA, compound **1** was successfully isolated using the guidance of positive staining of eastern blotting. Figure 2 shows TLC pattern using UV absorption and eastern blot profile for the standard solution of 3MGA, urine sample, and isolated compound **1** as a new GA metabolite. By ESIMS, ^1^H, ^13^C, and 2D NMR, and ROESY data in comparison with 18*β*-GA, compound **1** was identified as 22*α*-hydroxy-18*β*-glycyrrhetyl-3-*O*-sulfate-30-glucuronide (1) (Fig. 1) [14].

The discovery of compound **1** suggested that there might be other metabolites of GA as the causal candidates for licorice-induced pseudoaldosteronism. Compound **1** has a sulfate group at C-3, a hydroxyl group at C-22, and a glucuronic acid group at C-30. It is considered to be biosynthesized by three-step metabolic reactions via sulfotransferase (SULT), cytochrome P450 (CYP), and glucuronyl transferase. Therefore, it is predicted that there are other metabolites of GA that are biosynthesized by one- or two-step metabolic reactions among these three-step reactions. Further fractionation was performed for EHBR urine to isolate sulfate conjugates, and I isolated compound **2** as a new GA metabolite, and **3** that was first isolated from the bile of rats intravenously treated with GA by Jing et al. [15]. By ESIMS, ^1^H, ^13^C, and 2D NMR, and ROESY data in comparison with 18*β*-GA, compound **2** and **3** were identified as 22*α*-hydroxy-18*β*-glycyrrhetyl-3-*O*-sulfate (**2**) and 18*β*-glycyrrhetyl-3-*O*-sulfate (**3**) (Fig. 1) [16].

### Pharmacokinetics of compounds 1–3 and their possibilities as the causative compounds for pseudoaldosteronism

**Fig. 3:**
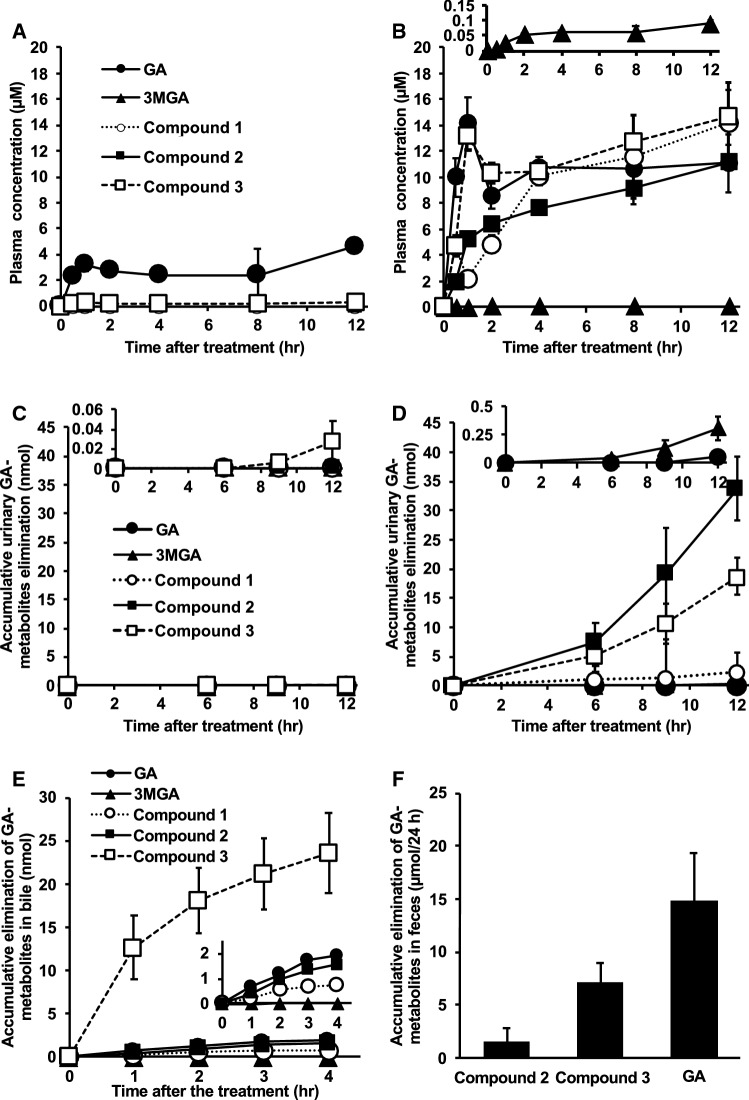
Pharmacokinetic profiles of GL metabolites in SD rats (**a**, **c**, **e**, **f**) and EHBRs (**b**, **d**) after the administration of GA [16]. GA (200 mg/kg) was administered orally to anesthetized SD rats or EHBRs, and plasma and urine were collected for 12 h (**a**–**d**). GA (0.2 mg/kg) was intravenously injected into anesthetized SD rats in which the biliary tract was cannulated. Then, bile samples were collected for 4 h (**e**). GA (0.2 mg/kg) was intravenously injected into conscious SD rats, and the feces was collected for 24 h. 3MGA and **1** were below detectable levels in the feces (**f**). Since the determined levels of 3MGA for B and D, **3** for C, GA for D, and all compounds except for **3** for E were relatively low; their magnified versions of their graphs are shown at the top or as an inside of each graph. The concentrations of GA metabolites were measured by LC–MS/MS, and data are plotted as mean ± S.E. (*n* = 4 for A–D, F; *n* = 3 for E)

I successively collected plasma and urine samples from both female SD rats and EHBRs orally administered GA and measured the concentrations of GA, 3MGA, and compounds **1**–**3** by LC–MS/MS. Figure 3a, c show the plasma concentration profiles and urinary elimination of GA and its metabolites in SD rats, respectively; Figs. 3b, d show those in EHBRs. In SD rats, GA was appeared in the plasma at 30 min and peaked once 1 h after the oral treatment. Then, the profile of the plasma GA concentration exhibited a biphasic curve for 12 h. The concentration of GA in the plasma was 4.7 μM at 12 h. The only other metabolite to appear in plasma was compound **3**, which was present at a concentration of 0.3 μM at 12 h. In the urine of SD rats, 0.03 nmol **3** was detected in the accumulated urine collected 12 h after the oral treatment, although GA, 3MGA, compounds **1**, and **2** were below detectable levels [16].

In the plasma of EHBRs treated with GA, the maximum concentration of GA occurred 1 h after the treatment. The concentration of GA was decreased at 2 h and increased again at 4 h, after which it was maintained for 12 h. Although the concentration of compound **3** at 30 min was approximately half that observed for GA, a plasma concentration profile similar to that for GA appeared after that. Compounds **2** and **1** appeared in the plasma and their concentrations gradually increased for 12 h. The concentrations of GA and compounds **1**–**3** at 12 h after treatment were at approximately similar and all were more than 100-fold greater than that of 3MGA (0.090 μM). In the urine of EHBRs treated with GA, 3MGA and compounds **1**–**3** were gradually eliminated, with the levels of **1**, **2**, and **3** in the accumulated urine for 12 h being 8-, 110-, and 62-fold of that of 3MGA (0.31 nmol), respectively. GA was detected in the urine at low levels (0.05 nmol).

GA, 3MGA, and compounds **1**–**3** were gradually excreted into the bile in SD rats intravenously injected with GA and the accumulation of compound **3** in the bile at 4 h was 12-, 1.5 × 10^3^-, 32-, and 15-fold those of GA, 3MGA, compounds **1**, and **2**, respectively (Fig. 3e). I confirmed that **3** was the major metabolite of GA eliminated into the bile in SD rats.

GA, compounds **3**, and **2** were found in the feces of SD rats collected for 24 h after the intravenous injection of GA, while 3MGA and compound **1** were not found in the feces (Fig. 3f) [16].

As predicted that in human blood samples compound **3** would be produced via a metabolic reaction from GA via a type of SULTs in the liver, I prepared an in vitro metabolic reaction system using a commercial fraction of human liver cytosol and recombinant SULTs. Compound **3** was produced from GA in the human liver cytosol fraction with a Km value (Michaelis constant) of 0.61 ± 0.44 μM (mean ± S.E., repeated four times) based on Hanes–Woolf plots. GA was not metabolised to compound **3** by SULT1A1 and 2B1, but SULT2A1 metabolised GA with a Km value of 0.73 ± 0.28 μM (mean ± S.E., repeated four times) [17].

These results suggest that the order of GA metabolism would be 3-*O*-sulfate conjugation by SULT2A1, 22-hydroxylation by CYP, and then 30-glucuronic acid conjugation by glucuronyl transferase in the liver. Under normal conditions, compound **3** may soon be eliminated from the liver into the bile via Mrp2, where the concentrations of compounds **2** and **1** in the bile of SD rats injected intravenously with GA were much lower than that of compound 3. Since we could detect compound **3** in the bile and GA in the feces of SD rats intravenously treated with GA, compound **3** would be hydrolyzed into GA by the enteric bacteria and reabsorbed into the circulation via enterohepatic circulation. This was supported by the biphasic profile of plasma GA concentration in SD rats orally treated with GA (Fig. 3a). Finally, GA would be eliminated into the feces as GA or compound **3**. On the other hand, the concentrations of 3MGA in the plasma and the urine of EHBRs orally treated with GA were much lower than those of compounds **1**–**3**, revealing that 3MGA is a minor metabolite of GA in EHBRs.

In rat serum, compounds **1**–**3** were present as the binding-form to albumin at a rate of 99.9% or more, so that they are difficult to be excreted by glomerular filtration.

Rat kidney slices were prepared from SD rats and incubated with the pooled plasma collected from EHBRs orally treated with GA for 2 h. The uptakes of compounds **1**–**3** by the kidney slices incubated at 37 °C were significantly higher than those incubated at 4 °C, although the uptakes of GA were not different in the samples incubated at 4 °C or 37 °C. It is suggested that compounds **1**–**3** were transported into tubular cells actively.

Madin-Darby canine kidney (MDCK) II cells stably expressing OAT1, 3, or mock cells were incubated with pooled plasma collected from EHBRs orally treated with GA for 15 min. Although the uptake of GA into the cells expressing OAT1 or 3 was approximately the same as that into mock cells, the uptake of compounds **1**–**3** into the cells expressing OAT1 or 3 was significantly higher than those into mock cells. It is suggested that compounds **1**–**3** can be transported into the cells actively via OAT1 and 3, and eliminated into the urine by tubular secretion [14, 16].

**Fig. 4:**
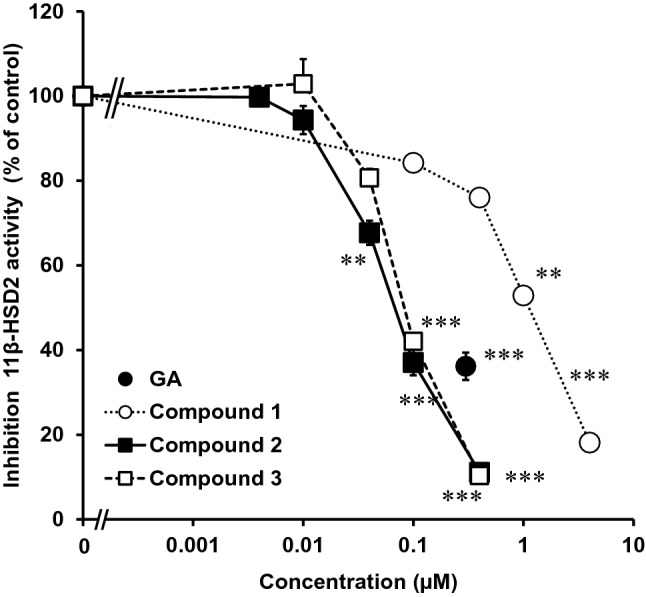
Inhibitory effects of compounds **1**–**3** on 11*β*-HSD2 using rat kidney microsome [14; 16]. [^3^H] cortisone and each compound were mixed with the rat kidney microsome fraction, and incubated at 37 °C for 30 min. Then, the amount of [^3^H] cortisol was measured. Data are expressed as mean ± S.E. (*n* = 4) of the percentage relative to the amount of [^3^H] cortisol in the mixture without samples. ** *p* < 0.01 and *** *p* < 0.001 compared with the groups without the samples by Dunnett’s multiple *t* test for compounds **1**–**3**, and by Student’s *t* test for GA

The IC_50_ values of 11*β*-HSD2 inhibitory activity of compounds **1**–**3** using rat kidney microsomes were 2.0, 0.11 and 0.10 µM, respectively (Fig. 4) [14, 16]. Those values of 3MGA, GA, and GL were 0.25, 0.31, and 1.8 μM, respectively [12]. The present results suggest that compounds **1**–**3**, rather than GA and 3MGA, can inhibit 11*β*-HSD2 to cause pseudoaldosteronism in vivo because 11*β*-HSD2 is existed in the microsomes of tubular cells [18] and the concentration of 3MGA was much lower than those of compounds **1**–**3**.

### Concentrations of GL metabolites in the plasma of a patient with pseudoaldosteronism

The patient was a 76-year-old female who had taken a Kampo formula containing licorice for 3 years (1.5 g of licorice for 1 year and 2 months, and 3 g for 1 year and 10 months). On day 0, we found a low plasma potassium level (2.1 mEq/l) and a high creatinine kinase level (364 U/l). Since low plasma renin activity and a low aldosterone level were also found, the patient was diagnosed with pseudoaldosteronism due to licorice. The administration of the Kampo formula was stopped and potassium supplementation of up to 100 mEq/day was started. In the plasma collected on day 0, we detected compound **3** at 8.6 μM, GA at 1.3 μM, and compound **2** at 87 nM, while compound **1**, GL and 3MGA were not detected. On day 5, the plasma potassium level was still 2.1 mEq/l, and potassium supplementation was continued. However, the plasma concentrations of compound **3** and GA had decreased to 3.6 μM and 0.65 μM, respectively. Compounds **2**, **1**, GL, and 3MGA were not detected. On day 13, the plasma potassium level was increased to 2.8 mEq/l, and the concentrations of compound **3** and GA were 61 nM and 11 nM, respectively. On day 14, the plasma potassium level was 3.4 mEq/l, and the concentration of compound **3** was 57 nM; GA was not detected. On day 18, the plasma potassium level had recovered to a normal level (4.9 mEq/l), so potassium supplementation was stopped. On this day, the concentration of compound **3** was below the detectable limit [16].

A multicenter retrospective study was conducted to clarify the association between the concentration of GL metabolites and the development of pseudoaldosteronism using the serum and urine of patients who took Kampo prescriptions containing licorice. A total of 97 patients were enrolled (age 60 ± 15 years, male/female 14:83). Among these, 67 had GA detected in serum (median 122 nM, 5 nM–1.8 µM) and 68 had compound **3** (median 239 nM, 2 nM–4.2 µM). There were no cases of detection of GL, 3MGA and compounds 1 and 2. A strong positive correlation (*r*^*2*^ = 0.80) between serum concentrations of GA and compound 3 was found, and the concentration of compound **3** was approximately twofold higher than that of GA, suggesting that compound **3** was identified as the major GL metabolite in human serum. No correlation was found between compound **3** and GA concentrations and blood pressure and edema. High blood compound **3** levels were associated with low plasma renin activity, plasma aldosterone levels, and serum potassium levels. It is suggested that compound **3** would be the causative agent of licorice-induced pseudoaldosteronism in human [17].

### Detection of compound 3 using anti-3MGA-mAb

**Fig. 5:**
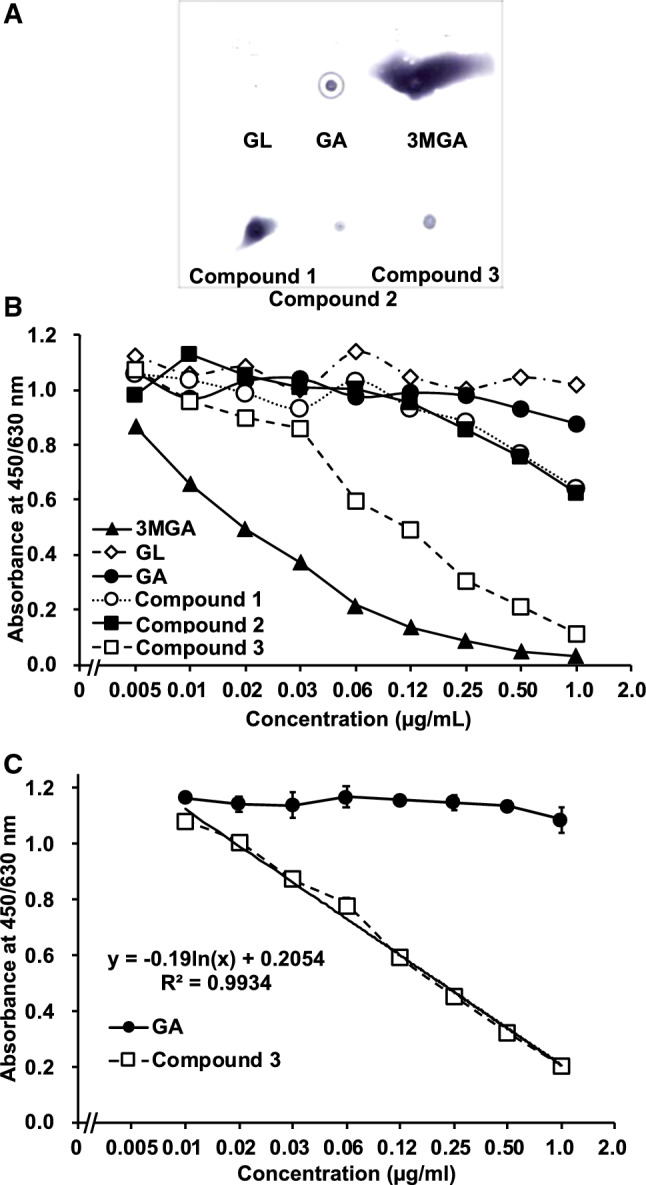
Dot-blot analysis and ELISA detection range of GL metabolites using anti-3MGA-monoclonal antibody (mAb) [16]. GL metabolites (1 μg each) were spotted on a PES membrane, and colored using an anti-3MGA-mAb (**a**). Competitive ELISA using anti-3MGA-mAb for GL metabolites was performed. Data are represented from a single analysis (*n* = 1) (**b**). Competitive ELISA using an anti-3MGA-mAb for compound **3** and GA was performed. Data are represented as mean ± S.D. (*n* = 3). Two-way ANOVA indicated a significant difference between GA and compound **3** (F_1,36_ = 4441, *p* < 0.001), concentrations (F_8,36_ = 315, *p* < 0.001), and their interactions (F_8,36_ = 281, *p* < 0.001). The calibrated line for the absorbance-concentration profile of compound **3** was calculated by the linear least-squares method, and the regression formula is shown in graph (**c**)

We confirmed the cross-reactivity of anti-3MGA-mAb against these GL metabolites. GL, GA, 3MGA, and compounds **1**–**3** (1 μg each) were spotted on a PES membrane, fixed onto the membrane, and colored using an anti-3MGA-mAb (Fig. 5a). By imaging analysis, the areas of positive staining (in pixels) were follows: GL, not detectable; GA, 97; 3MGA, 3395; compound **1**, 606; **2**, 79; and **3**, 146. Thus, the anti-3MGA-mAb cross-reacted to some extent with other metabolites of GL [16].

Figure 5b shows the concentration profiles of GL metabolites and absorbance in a competitive ELISA system by using anti-3MGA-mAb. When the specificity of the mAb to 3MGA was calibrated to 100%, the cross-reactivities for GL, GA, compounds **1**–**3** were 0.23%, 2.2%, 4.8%, 4.0%, and 23%, respectively. By the result of spot test, the cross-reactivity of anti-3MGA-mAb with compound **1** was higher than that with compound 3 (Fig. 5a), this order was inverse in the result of ELISA, since the binding of the compound **1** onto PES membrane would be higher than that of compound **3**. As mentioned above, compound **3** is suggested as the causative agent of pseudoaldosteronism in humans, where the plasma of patients taking licorice mainly contains both compound **3** and GA. We confirmed the selectivity of the anti-3MGA-mAb in discriminating between compound **3** and GA, and established the detectable measurement range for compound **3** in this ELISA system. Two-way ANOVA revealed that the anti-3MGA-mAb had significant selectivity for compound **3** compared with GA (*p* < 0.001), and the detectable concentration range for compound **3** was 7.8 ng/ml (14 nM) to 1.0 μg/ml (1.8 μM) (Fig. 5c). It may be possible to detect compound **3** in the plasma or the urine of patients with sufficient sensitivity through competitive ELISA using anti-3MGA-mAb [16].

## Conclusion

**Fig. 6:**
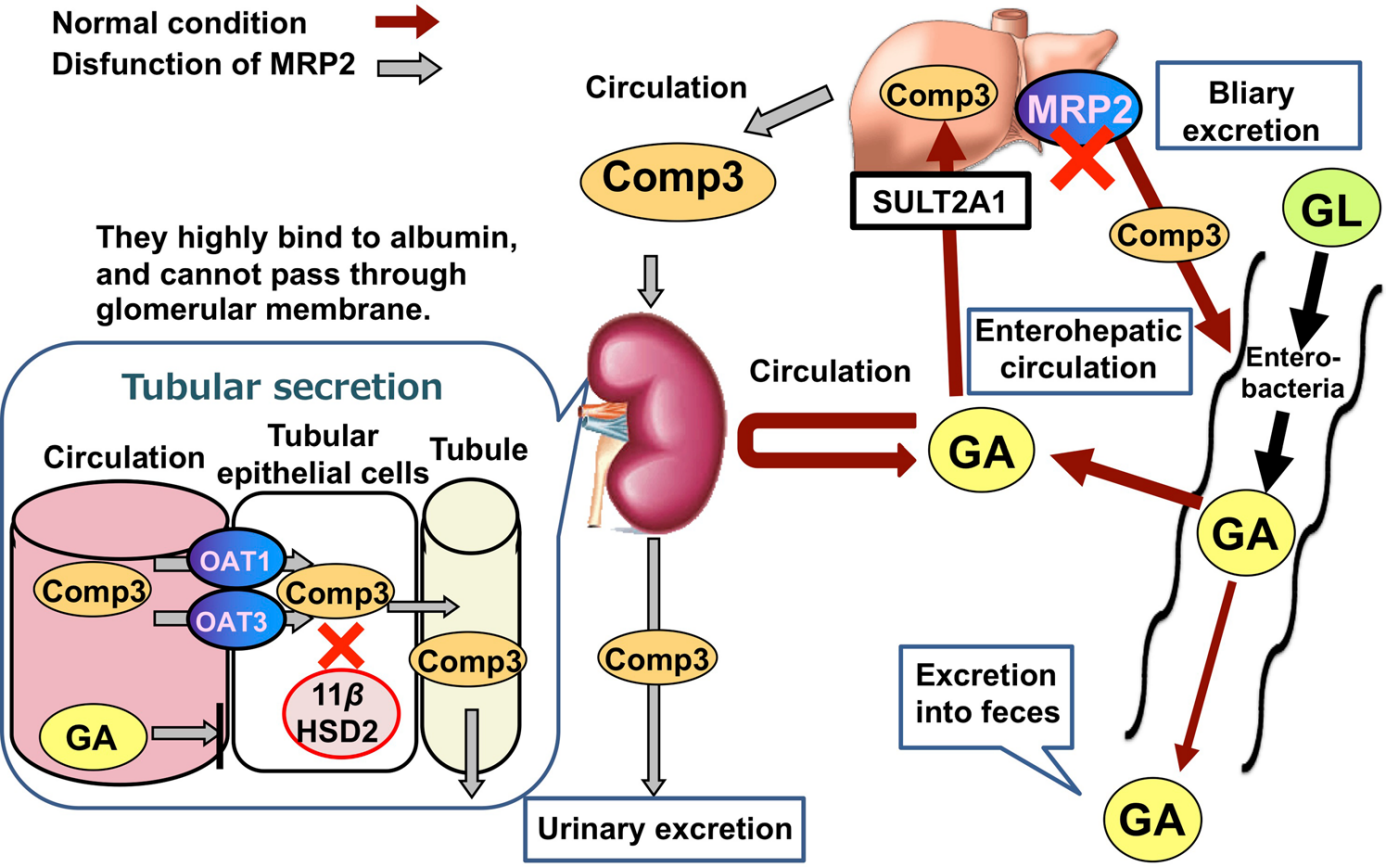
Schematic diagram of the pharmacokinetics of glycyrrhizin (GL) and its metabolites after oral administration of GL in human. GA, glycyrrhetinic acid; Comp3, 18*β*-glycyrrhetyl-3-*O*-sulfate (**3**); MRP, multidrug resistance-related protein; OAT, organic anion transporter; 11βHSD2, type 2 11*β*-hydroxysteroid dehydrogenase

In human, when GL is orally administered, GL is hydrolyzed to GA by intestinal bacteria, and then absorbed into blood circulation. GA in blood circulation is not excreted into urine and transfers into liver, metabolized to compound **3** by SLUT2A1, and excreted into bile via Mrp2. In intestine, compound **3** is hydrolyzed to GA again by intestinal bacteria, and is partially absorbed again from the intestine into blood circulation to exhibit enterohepatic circulation, and the unabsorbed portion of compound **3** is excreted in feces. When bile excretion of compound **3** is suppressed due to Mrp2-dysfunction, compound **3** is transferred into blood circulation. Since compound 3 is existed in blood circulation with the binding-form to serum albumin, compound **3** is not excreted into urine by glomerular filtration. However, it can be transported from blood circulation into tubular cells via OAT1 and 3, and can inhibit 11*β*HSD2 to develop pseudoaldosteronism (Fig. 6).

Therefore, when using Kampo medicines containing licorice, it may be possible to diagnose the predisposition to develop pseudoaldosteronism by detecting compound **3** in blood or urine at an early stage. In addition, GA cannot be transferred to renal tubular epithelial cells because the most of GA is bound to albumin in the blood, but during hypoalbuminemia, the concentration of free GA may increases and transport into tubular cells by passive diffusion to inhibit 11*β*HSD2 [19, 20]. On the other hand, the concentration of urinary compound **3** in patient samples was lower than that in blood concentration [17]. Therefore, it is undeniable that unknown GL metabolites that could not be detected may exist in humans. I would like to continue my research in the future.

## References

[1] United States Pharmacopeial Convention (2020). United Sates Pharmacopeia 43–National Formulary 38.

[2] Pharmaceutical and Medical Device Regulatory Science Society of Japan (2016). Japanese pharmacopoeia seventeenth edition (JP XVII).

[3] Japan Kampo Medicines Manufacturers’ Association under the super vision of National Institute of Health Sciences, the affiliated institutions of the Ministry of Health, Labour and Welfare of Japan (2013). Handbook on OTC medicinal products in Kampo.

[4] Conn J, Rovner D, Cohen E (1968). Licorice-induced pseudoaldosteronism. Hypertension, hypokalemia, aldosteronopenia, and suppressed plasma renin activity. JAMA.

[5] Morimoto Y, Nakajima C (1991). Pseudoaldosteronism induced by licorice derivatives in Japan. J Trad Med.

[6] Homma M, Ishihara M, Qian W, Kohda Y (2006). Effects of long term administration of Shakuyaku-kanzo-To and Shosaiko-To on serum potassium levels. Yakugaku Zasshi.

[7] Akao T, Hayashi T, Kobashi K, Kanaoka M, Kato H, Kobayashi M, Takeda S, Oyama T (1994). Intestinal bacterial hydrolysis is indispensable to absorption of 18*β*-glycyrrhetic acid after oral administration of glycyrrhizin in rats. J Pharm Pharmacol.

[8] Edwards CR (1990). Renal 11*β*-hydroxysteroid dehydrogenase: a mechanism ensuring mineralocorticoid specificity. Hormone Res.

[9] Monder C, Stewart PM, Lakshmi V, Valentino R, Burt D, Edwards CR (1989). Licorice inhibits corticosteroid 11*β*-dehydrogenase of rat kidney and liver: *in vivo* and *in vitro* studies. Endocrinology.

[10] Kato H, Kanaoka M, Yano S, Kobayashi M (1995). 3-Monoglucuronyl-glycyrrhetinic acid is a major metabolite that causes licorice-induced pseudoaldosteronism. J Clin Endocrinol Metab.

[11] Makino T, Ohtake N, Watanabe A, Tsuchiya N, Imamura S, Iizuka S, Inoue M, Mizukami H (2008). Down-regulation of a hepatic transporter multidrug resistance-associated protein 2 is involved in alteration of pharmacokinetics of glycyrrhizin and its metabolites in a rat model of chronic liver injury. Drug Metab Dispos.

[12] Makino T, Okajima K, Uebayashi R, Ohtake N, Inoue K, Mizukami H (2012). 3-Monoglucuronyl-glycyrrhretinic acid is a substrate of organic anion transporters expressed in tubular epithelial cells and plays important roles in licorice-induced pseudoaldosteronism by inhibiting 11 beta-hydroxysteroid dehydrogenase 2. J Pharmacol Exp Ther.

[13] Makino T (2014). 3-Monoglucuronyl glycyrrhetinic acid is a possible marker compound related to licorice-induced pseudoaldosteronism. Biol Pharm Bull.

[14] Morinaga O, Ishiuchi K, Ohkita T, Tian C, Hirasawa A, Mitamura M, Maki Y, Yasujima T, Yuasa H, Makino T (2018). Isolation of a novel glycyrrhizin metabolite as a causal candidate compound for pseudoaldosteronism. Sci Rep.

[15] Jing J, Ren W, Chen X, Wang Y, Yu Q, Wang G, Davey AK, Wang J, Jing N (2008). Glucuronide-sulfate diconjugate as a novel metabolite of glycyrrhetic acid in rat bile. Drug Metab Pharmacokinet.

[16] Ishiuchi K, Morinaga O, Ohkita T, Tian C, Hirasawa A, Mitamura M, Maki Y, Kondo T, Yasujima T, Yuasa H, Minamizawa K, Namiki T, Makino T (2019). 18*β*-glycyrrhetyl-3-*O*-sulfate would be a causative agent of licorice-induced pseudoaldosteronism. Sci Rep.

[17] Takahashi K, Yoshino T, Maki Y, Ishiuchi K, Namiki T, Ogawa-Ochiai K, Minamizawa K, Makino T, Nakamura T, Mimura M, Watanabe K (2019). Identification of glycyrrhizin metabolites in humans and of a potential biomarker of liquorice-induced pseudoaldosteronism: a multi-centre cross-sectional study. Arch Toxicol.

[18] Odermatt A, Kratschmar DV (2011). Tissue-specific modulation of mineralocorticoid receptor function by 11beta-hydroxysteroid dehydrogenases: an overview. Mol Cell Endocrinol.

[19] Shimada S, Arai T, Takmaoka A, Homma M (2016). Hypoalbuminemia is a risk factor for hypokalemia due to yokukansan preparations. Jpn J Clin Pharmacol Ther.

[20] Komatsu A, Yoshino T, Suzuki T, Nakamura T, Kanai T, Watanabe K (2019). Risk factors associated with pseudoaldosteronism in patients with chronic hepatitis: a retrospective cohort study. Basic Clin Pharmacol Toxicol.

